# Comparative Genomic Analysis Reveals Intestinal Habitat Adaptation of *Ligilactobacillus* *equi* Rich in Prophage and Degrading Cellulase

**DOI:** 10.3390/molecules27061867

**Published:** 2022-03-14

**Authors:** Yu Li, Chen Liu, Qing Liu, Wenjun Liu

**Affiliations:** 1 Key Laboratory of Dairy Biotechnology and Engineering, Ministry of Education, Inner Mongolia Agricultural University, Hohhot 010018, China; ly401337962@163.com (Y.L.); lc911005xz@163.com (C.L.); liuqing0471@126.com (Q.L.); 2 Key Laboratory of Dairy Products Processing, Ministry of Agriculture and Rural Affairs, Inner Mongolia Agricultural University, Hohhot 010018, China; 3 Inner Mongolia Key Laboratory of Dairy Biotechnology and Engineering, Inner Mongolia Agricultural University, Hohhot 010018, China; 4 Collaborative Innovative Center of Ministry of Education for Lactic Acid Bacteria and Fermented Dairy Products, Inner Mongolia Agricultural University, Hohhot 010018, China

**Keywords:** comparative genomics, carbohydrate-active enzymes, *Ligilactobacillus equi*

## Abstract

*Ligilactobacillus* *equi* is common in the horse intestine, alleviates the infection of *Salmonella*, and regulates intestinal flora. Despite this, there have been no genomic studies on this species. Here, we provide the genomic basis for adaptation to the intestinal habitat of this species. We sequenced the genome of *L.* *equi* IMAU81196, compared this with published genome information from three strains in NCBI, and analyzed genome characteristics, phylogenetic relationships, and functional genes. The mean genome size of *L. equi* strains was 2.08 ± 0.09 Mbp, and the mean GC content was 39.17% ± 0.19%. The genome size of *L. equi* IMAU81196 was 1.95 Mbp, and the GC content was 39.48%. The phylogenetic tree for *L. equi* based on 1454 core genes showed that the independent branch of strain IMAU81196 was far from the other three strains. In terms of genomic characteristics, single-nucleotide polymorphism (SNP) sites, rapid annotation using subsystem technology (RAST), carbohydrate activity enzymes (CAZy), and predictions of prophage, we showed that strain *L. equi* JCM 10991^T^ and strain DSM 15833^T^ are not equivalent strains.It is worth mentioning thatthestrain of *L. equi* has numerous enzymes related to cellulose degradation, and each *L. equi* strain investigated contained at least one protophage. We speculate that this is the reason why these strains are adapted to the intestinal environment of horses. These results provide new research directions for the future.

## 1. Introduction

*Lactobacillus* was reclassified into 25 genera in 2020, which included the *Lactobacillus delbrueckii* group, *Paralactobacillus*, and 23 new genera. *Ligilactobacillus* is one of the new genera that form a specific subgroup adapted to different ecological habitats [1]. The *Ligilactobacillus* genus has 16 species, including *Ligilactobacillus* *animalis*, *Ligilactobacillus* *ruminis*, *Ligilactobacillus* *acidipiscis*, and *Ligilactobacillus* *agilis*. Most *Ligilactobacillus* strains were isolated from animal and human gastrointestinal tracts and adapted to the gut environment of vertebrate hosts. Several *Ligilactobacillus* species have also been isolated from fermented food and used as starter cultures or probiotics [2,3]. There is a symbiotic relationship between intestinal microorganisms and the host. The gut environment and eating habits of the host have an impact on intestinal flora. Furthermore, the host’s physiological activities involve intestinal microorganisms [4]. In previous studies, some strains of the genus *Ligilactobacillus* have been shown to have prebiotic effects, such as contributing to the regulation of intestinal flora [5], alleviation of colitis [6], and high antibacterial activity [7]. However, there are few reports of these functions for *L. equi*.

*L. equi* is a lactic acid bacterium that was first isolated in 2002 from the feces of horses in Hokkaido, Aomori-ken, Chiba-shi, and elsewhere in Japan [8]. It is the predominant bacterial species in healthy horse intestines [8]. One study of strains from horse feces samples showed that all samples contained *Ligilactobacillus* *hayakitensis*, *Limosilactobacillus* *equigenerosi*, and *L. equi* [9]. As *L. equi* is the dominant species isolated from the intestine of horses, we speculate that it is likely to be specifically adapted to the environment of the intestinal tract of horses.

Comparative genomics is used to compare the genomes of members of the same species or individuals from different species. It can reveal changes that occur during the evolution of a species and clarify the evolutionary relationship between species and the internal structure of the species’ genome [10]. In-depth studies of physiological and metabolic mechanisms at the genetic level can help improve a species’ production and utilization value [11]. Genomic analysis of *L. acidipiscis* ACA-DC 1533 from traditional cheese showed thatglycine-betaine was present in this strain, and the gene was related to the growth ability of the strain in fermented foods with a high salt concentration [12]. In a study of the genomes of *L. rumin* is strains from different ecological sites, it was found strains were adapted to different host intestinal environments [13]. Identification of enzymes (such as glycan hydrolase) in the draft genome of *L. equi* DPC 6820 shows that this strain is adapted to the gastrointestinal tract of herbivorous animals such as horses [14]. As of May 2021, the NCBI website has only published genomic information for three strains of *L. equi*, and genetic research on this species is lacking. To the best of our knowledge, there have been no comparative genomics studies on *L. equi*.

For these reasons, we used Illumina Novaseq next-generation sequencing technology to sequence the whole genome of *L. equi*IMAU81196. This strain was isolated from foal manure collected in Hongyuan County, Sichuan province, in 2014. This has greatly expanded our knowledge of the genetic background and genomic characteristics of *L. equi*. As the dominant bacterial species in the intestine of horses, *L. equi* may have intestinal adaptation mechanisms. By analyzing carbohydrate-active enzymes of four strains, we found that all contained enzymes related to the degradation of cellulose, and these enzymes are more conducive to the digestion and absorption of feed [15]. The use of comparative genetics to study *L. equi* at the genetic level provides a theoretical basis for the subsequent development and utilization of strains.

## 2. Results and Discussion

### 2.1. L. equi Genome Characteristics

The genome size of *Ligilactobacillus* was between 1.33 Mbp and 2.22 Mbp, and the mol GC content was between 32.50% and 43.37%. The numbers of predicted coding genes varied between 1273 (*Ligilactobacillus* *ceti* DSM 22408^T^) and 2425 (*L. equi* JCM 10991^T^). The genome size of *L. equi* was 2.08 ± 0.09 Mbp, and the DNA GC content was 39.17 ± 0.19%. Among the four strains of *L. equi*, strain IMAU81196 had the smallest genome (1.95 Mbp) and the largest GC content (39.5%). All genome information is shown in Table 1. Strain DSM 15833^T^ and strain JCM 10991^T^ were equivalent strains, but statistical analysis of genome information showed that these two strains were different in genome size, GC content, CDs, and tRNA.

From the perspective of genomic characteristics, *L. equi* was similar to other strains in the genus *Ligilactobacillus*. According to the information we currently know, *L. equi* is only isolated from the intestine of horses. Studies have also shown that *L. equi* is the dominant strain in the intestine of horses [9]. Some previous work shows that lactic acid bacteria are associated with various ecological loci. In one study on the adaptive lifestyle of *L. ruminis*, differences in niches had the greatest impact on the evolution of bacterial genes [13]. Lactic acid bacteria simplify their metabolic processes in order to adapt to their living environment during the evolution process, and genes in these strains will be lost. Some strains have lost many ancestral genes during evolution, which gradually reduces the strain’s genome size [16].

### 2.2. Analysis of the Average Nucleotide Identity and Total Nucleotide Identity of Ligilactobacillus Type Strains

**Figure 1:**
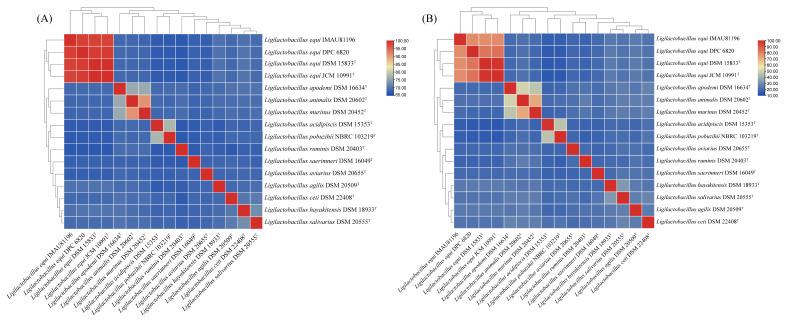
ANI analysis (**A**) and TNI analysis (**B**) of type strains from the genus *Ligilactobacillus*.

Average nucleotide identity (ANI) is often used for species identification, and it is the gold standard for species calibration [17,18]. An ANI value of 95–96% is the boundary of species demarcation. Theoretically, strains with ANI values greater than 95% are regarded as the same species [19,20]. The results are shown in Figure 1: the clustering results displayed by the ANI and TNI values of the strains are consistent, but the ANI value has a higher resolution, and the relationship between the strains can be seen more clearly.

Analyzing the ANI value of the genus *Ligilactobacillus*, the strains showed that they could be divided into three clusters. The four strains of *L. equi*were clustered together. The three strains of *Ligilactobacillus apodemi*, *L*. *animalis*, and *Ligilactobacillus murinus* were grouped together. Among them, the ANI value of *L. animalis* and *Ligilactobacillus murinus* was 91.06%, and the ANI value of *L*. *apodemi* and the other two was 76%. This shows that the genetic distance between *L. animalis* and *Ligilactobacillus murinus* is small. The remaining strains could be classified as a branch, and the ANI value of these strains was about 67%. The TNI value of the four strains of *L. equi* was above 78%, and the ANI value of them was above 98%, so they can be regarded as the same species. The ANI value of IMAU81196 and DPC 6820 was 98.44%, which was the highest of all strains, indicating that the base and nucleic acid match between IMAU81196 and DPC 6820 was higher. The ANI value of strains JCM 10991^T^ and DSM 15833^T^ was 99.95%, indicating that there are differences between the homologous gene regions of these two strains.

### 2.3. Core Gene Set to Construct Phylogenetic Tree

**Figure 2:**
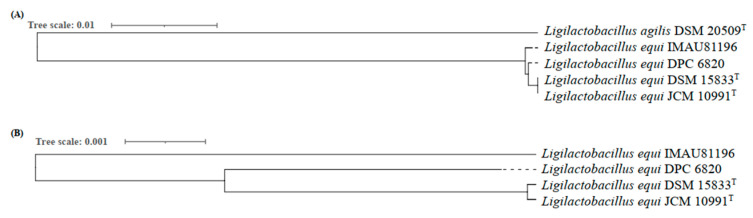
(**A**) Phylogenetic relationship among four strains of *L. equi* based on 247 core gene *L*. *agilis* DSM 20509 as outgroup and (**B**) phylogenetic relationship among four strains of *L. equi* based on 1454 core genes.

The phylogenetic tree uses branches to represent the genetic relationships among the studied species. It is mainly through DNA sequencing and protein sequencing that the evolutionary history of species can be inferred [21]. Based on 247 core genes, the phylogenetic tree of four strains of *L. equi* was constructed by neighbor-joining (NJ) (the strain type DSM 20509^T^ of strain *L. agilis* was used as the outgroup) (Figure 2A). A second phylogenetic tree of the four strains of *L. equi* was also constructed based on 1454 core genes (Figure 2B). We also constructed a phylogenetic tree of 16 strains from the genus *Ligilactobacillus* based on 97 core genes (Figure S1).

It can be seen from the results shown in Figure S1 that there is a close relationship between *L. equi* and *L. agilis*. Studies have shown that *L. agilis* is a species isolated from the gastrointestinal tract of birds, pigs, and other animals, while *L. equi* is a species only isolated from the intestinal tract of horses. *Ligilactobacillus pobuzihii* and *Ligilactobacillus acidipisis*, which are distant from both *L. equi* and *L. agilis*, are isolated from fermented foods such as soy sauce, vinegar, and fermented fish [21,22].

From the perspective of the phylogenetic tree, the evolutionary divergence time between *L. equi* IMAU81196 and *L. equi* DPC 6820 is shorter, and the genetic relationship is closer. The branches for strains JCM 10991^T^ and DSM 15833^T^ are longer, indicating that evolution was greater, and the phylogenetic relationship between the two strains was similar. According to NCBI, strain JCM 10991^T^ and strain DSM 15833^T^ are equivalent strains. According to our results and phylogenetic tree, we have shown that they are closely related and have the same degree of evolution. However, whether the two are equivalent strains still needs further verification.

### 2.4. Gene Prediction and Annotation

**Figure 3:**
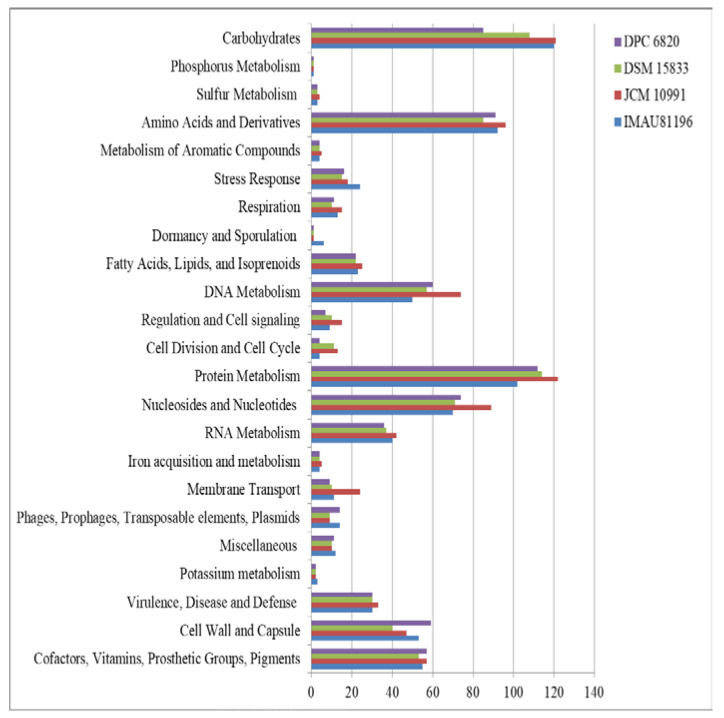
RAST annotation of four *L. equi* strains.

The results showed that a mean of 747 protein-coding genes was annotated in the four strains of *L. equi*, among which *L. equi* JCM 10991^T^ had the most genes (828), and the remaining three strains had about 720 genes. After annotation, 23 functional categories were obtained, including carbohydrates, amino acids and derivatives, DNA metabolism, protein metabolism, and metabolism of aromatic compounds, among others. In the genome of *L. equi*, the largest proportion of related genes encoded protein metabolism (15.05%), followed by genes encoding carbohydrates (14.51%), amino acids and their derivatives (12.17%), nuclear glycoside and nucleotides (10.16%), and DNA metabolism (8.06%). The genome of *L. equi* was rich in genes with a diversity of functions (Figure 3). There were differences in functional genes among strains. Genes involved in carbohydrate metabolism and protein metabolism were most abundant.

Carbohydrate metabolism genes varied among the strains (Figure 3). *L. equi* JCM 10991^T^ has the largest number of carbohydrate genes (121 related genes representing 14.61% of all genes). The fewest carbohydrate genes were found in *L. equi* DPC 6820 (85 genes representing 11.92% of all genes). Compared with other strains, strain JCM 10991^T^ had about 100 more genes. After comparative analysis, strain JCM 10991^T^ had 120 more genes than DSM 15833^T^, and the two strains were far apart in terms of the total number of genes. It can be seen from the figure that DSM 15833^T^ has fewer genes than JCM 10991^T^ in each gene category, and there are obvious differences in nucleosides, nucleotides, and membrane transport. This proves that JCM 10991^T^ and DSM 15833^T^ are not equivalent strains.

With further analysis, we found that strain JCM 10991^T^ has genes for the operation of the pentose phosphate pathway, but these were not found in strain DPC 6820. There are many differences in the utilization of monosaccharides between the two strains. Strain JCM 10991^T^ can utilize fructose and L-arabinose, while strain DPC 6820 contains genes for the catabolism of deoxyribose and deoxynucleosides (Table S1). To a certain extent, this shows that different *L. equi* strains have differences in carbohydrate utilization. Carbohydrate is the main source of metabolic energy for *Lactobacillus* species and is important for ecological adaptation [23]. The intestine is the main digestive organ of horses and other herbivorous animals. It mainly uses intestinal microbial fermentation to decompose and utilize carbohydrates. Intestinal microbes play a more prominent role in the digestion and utilization of nutrients and energy metabolism [24]. *L. equi* shows differences in carbohydrate utilization. We speculate that it may be an evolutionary adaptation to different intestinal environments.

### 2.5. Construction of the Core Gene Set, Pan-Gene Set, and Unique Gene Analysis of L. equi

#### 2.5.1. Construction of Pan-Core Gene Set

**Figure 4:**
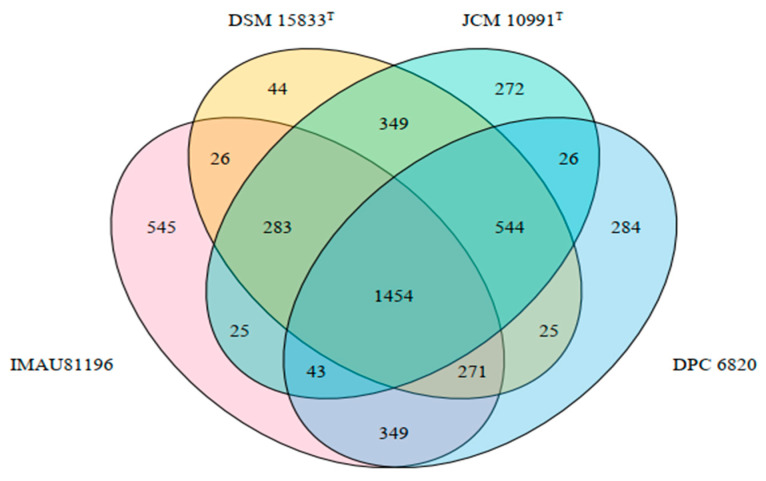
Venn diagram of common endemic genes of 4 strains of *L. equi*.

Through comparative genomics, using Prokka and Roary software to analyze the genetic differences among four strains of *L. equi*, we showed that the four strains had a total of 2995 pan-genes, including 1454 core genes and 1109 unique genes. *L. equi* IMAU81196 had the most unique genes with 545, *L. equi* DPC 6820 had 284 unique genes, *L. equi* JCM 10991^T^ had 272 unique genes, and *L. equi* DSM 15833^T^ had the least unique genes, with a total of 44 (Figure 4).

Excluding hypothetical genes and inserted sequences, IMAU81196 has 73 unique genes, DPC 6820 has 22 unique genes, JCM 10991^T^ has 102 unique genes, and DSM 15833^T^ has 1 unique gene. According to the results of specific gene analysis, the genes of JCM 10991^T^ and DSM 15833^T^ are quite different, and the two strains cannot be regarded as equivalent strains. The specific genes of different strains are quite different, which may be related to the isolation environment and the individual host differences. In a study on *Lactococcus*, it was found that the genomes of different strains within the genus were highly diverse [25]. This may be associated with the range of environments colonized by different *Lactococcus* species and the existence of numerous ways of exchanging genetic material. Strains make changes to adapt to the environment, and their genes also change accordingly [26]. Although the four strains of *L. equi* were all isolated from the intestinal tract of horses, the distance between the strains was relatively long, and the isolation environment was quite different, which may result in great differences in the genes of the strains.

#### 2.5.2. Analysis of Specific Functional Genes

In order to further analyze the differences between the strains, their unique genes were analyzed. IMAU81196 unique genes were mainly involved in the metabolism of carbohydrates. The *bgl*F, *bgl*H, *bgl*G, and *dha*MLK (PTS-dependent dihydroxyacetone kinase) genes encode the phosphoenolpyruvate (PEP)–glucose phosphotransferase system (PTS). Sugars are the main carbon and energy source for the growth of lactic acid bacteria. However, different strains have different carbohydrate utilization [27]. It is speculated that strain IMAU81196 has a strong ability to produce aromatic compounds and their derivatives. Analysis found that the strains have genes related to the shikimate pathway, such as *aro*A, *aro*C, and *saro*K (Table 2). The shikimic acid pathway is the main pathway for the synthesis of aromatic compounds. Strain IMAU81196 also has PEP (phosphoenolpyruvate) required for the initiation of the shikimate pathway, so we speculate that IMAU81196 has a strong ability to synthesize aromatic compounds.

The unique genes of strain JCM 10991^T^ were mainly involved in protein synthesis (e.g., *glt*X, *ala*S, *pro*S, *leu*S, *ile*S, *cyc*A), transmembrane transport (e.g., *fol*T, *znu*B, *nir*C), and other pathways. Glutamate-tRNA ligase, alanine-tRNA ligase, proline-tRNA ligase, and other aminoacyl-tRNA synthetases (*aar*S) are key enzymes in the protein synthesis pathway of organisms [28]. Its main function is to specifically recognize amino acid side chains and their corresponding tRNA [29]. One unique gene of strain DSM 15833^T^ is *fru*A (fructanbeta-fructosidase precursor), so the two strains should not be classified as equivalent strains.

### 2.6. Carbohydrate-Active Enzyme Analysis

CAZyme is a group of enzymes involved in the assembly and decomposition regulation of carbohydrate metabolism. It has the functions of degradation, modification, and generation of glycosidic bonds [30]. Carbohydrate-active enzymes are divided into six functional categories. The CAZy annotation results show that the four strains were annotated into four functional categories and 30 functional subcategories. The main enzymes were glycoside hydrolases (GHs) and glycosyltransferases (GTs).

**Figure 5:**
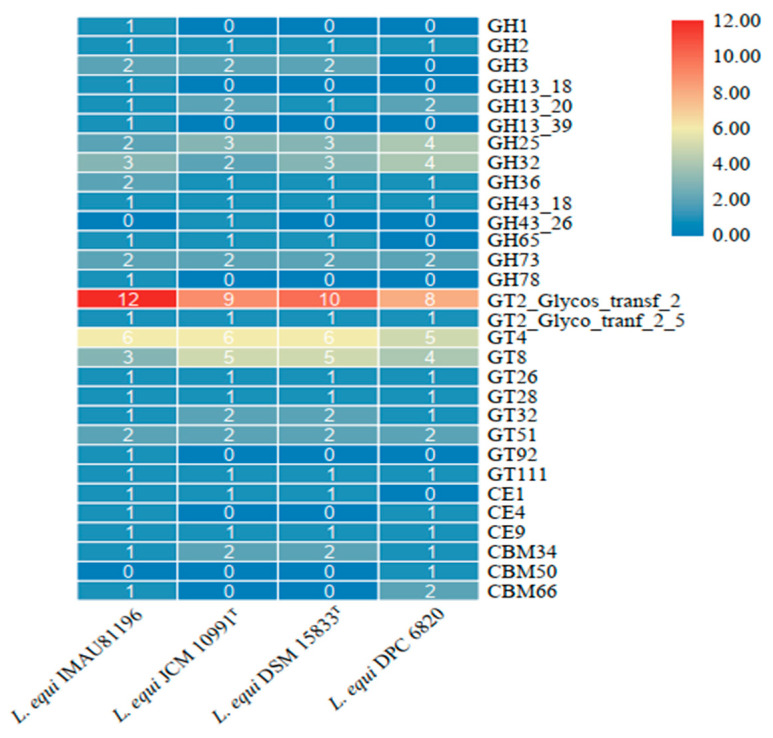
Gene number of CAZymes in four strains of *L. equi*.

The GTs family of enzymes was the most abundant in all four strains of *L. equi* (Figure 5). Among these were GT2, GT4, and GT8, which are three groups within GTs with a large number of enzymes. The GTs family is mainly responsible for the formation of glycosidic bonds. There are many types of enzymes in the GHs family, which are mainly responsible for hydrolysis and rearrangement of glycosidic bonds. The CE family performs ester hydrolysis of carbohydrates, while the CBM family is attached to carbohydrates [31]. The GH32 content is higher in the GHs family. GH32 encodes fructan β-(2,6)-fructosidase/6-exohydrolase (EC 3.2.1.154), sucrose 1-fructosyltransferase (EC 2.4.1.99), and another 14 kinds of enzymes. They are responsible for the hydrolysis and synthesis of fructan glycosidic bonds, including inulinase, sucrase, fructanase, and other hydrolase enzymesand fructosyl transferases [32]. Fructan selectively promotes the growth of *Bifidobacteria* and lactic acid bacteria in the intestine [33]. The GT2 family of enzymes encodes the largest number of enzymes, and Glycos_transf_2 has the largest number of copies in the GT2 family. This is a diverse family, transferring sugar from UDP-glucose, UDP-*N*-acetyl-galactosamine, GDP-mannose, or CDP-abequose to a range of substrates, including cellulose, dolichol phosphate, and teichoic acids. Xylan 1,4-β-xylosidase (EC 3.2.1.37) encoded by the GH3 family, encoded xylanase (EC 3.2.1.8) encoded by GH43, and acetyl xylan esterase (EC 3.1.1.72) encoded by the CE4 family are all hemicellulose degradation enzymes needed for the above process [34,35]. Based on these results, we speculate that *L. equi* may have the ability to degrade hemicellulose.

This may be related to the source of the strain. The strains of *L. equi* were all isolated from the intestine of horses, which are herbivores. Strains may adapt to the intestines of herbivorous animals and have the ability to degrade otherwise indigestible cellulose and hemicellulose in feed. The animal’s gastrointestinal tract will form a unique intestinal flora during the long-term interaction between food and the gut environment. Due to various factors such as diet, environment, genetics, etc., harmony among intestinal microbes has developed through evolution, and genes encoding corresponding enzymes have developed to adapt to the herbivorous lifestyle. *L. equi* is the dominant species in the intestine of horses and may have the ability to degrade cellulose and hemicellulose. We speculate that *L. equi* can promote the health of horses by increasing the availability of nutrients from feed, and that *L. equi* is specifically adapted to the intestines of horses.

### 2.7. Identification of the Presence of Prophages in L. equi

With the in-depth development of research technology, prophages and their residues are found in many bacterial genomes. The term prophage refers to the entire set of phage DNA genomes present in lysogenic bacteria [36]. Increasing numbers of studies have shown that the bacterial genome carries prophages, and some prophage sequences are close to 20% of the capacity of the bacterial genome [37]. The predicted prophages were classified into three categories: ‘intact’, ‘incomplete’, or ‘questionable’. A total of five complete prophages were identified from the four strains of *L. equi.*

There was a complete prophage region in each strain. Based on the analysis of the predicted results of DSM 15833^T^ and JCM 10991^T^, strain JCM 10991^T^ was identified as a complete prophage region (Table 3), and the most common phage was found in the genus *Lactobacillus*. In strain DSM 15833^T^, we identified two complete phages, one from *Lactobacillus* and the other from *Listeria*. This shows that DSM 15833^T^ and JCM 10991^T^ are not equivalent strains.

Bacteria and bacteriophages are most abundant in mammalian intestines. Bacteriophages are usually two orders of magnitude more abundant than bacteria. Due to the interactions among many factors in the intestine, the number of phages in the intestine varies [38]. Studies have shown that the structure and composition of phages depend on the physiological or pathological state of the host [39]. For example, during the growth of infants, the composition of phages changes greatly, and the composition of intestinal phages increases in stability with age [40]. At the same time, some studies have shown that phage characteristics in people with a similar dietary structure are more similar [41]. Ventura et al. reported that the *Lactiplantibacillus plantarum* genome contained four prophage elements [42]. The results of Shuo et al. showed that 44% of *L. ruminis* contained intact prophages [43]. Each strain of *L. equi* in this study contained prophages. We speculate that the possession of prophages is the result of intestinal adaptation.

### 2.8. Synteny Analysis

Synteny analysis is used to study the correlation between genes from different species. During divergence of strains from the same ancestor events such as genome rearrangement, gene horizontal transfer, and gene deletion often occur [44]. However, most genes are conserved, and the relative order of ancestral genes is maintained [45]. The size of the collinearity fragment is related to the evolution time. A larger fragment indicates that the strain has a shorter differentiation time and less accumulation of variation. In contrast, a longer differentiation time results in more variation and fewer common features [46].

Our results show that the four strains of *L. equi* have poor collinearity (Figure S2). Strains DSM 15833^T^, DPC 6820, and IMAU81196 have all undergone large-scale genome rearrangement, and phenomena such as inversion, insertion, and deletion are obvious. It shows that the strain has a longer evolution time and more genetic variation. This may be because the strains are derived from animal intestines, and the environment is complex. Under the pressure of long-term selection, local collinear regions of strains changed in terms of number, direction, arrangement sequence, and length to better adapt to the living environment. The results of RAST annotation, specific gene analysis, and prephage analysis showed that there were differences between the genomes of strain JCM 10991^T^ and strain DSM 15833^T^, which could not be identified as equivalent strains, so we carried out collinearity analysis. Strains JCM 10991^T^ and DSM 15833^T^ had poor collinearity and similarity, and DSM 15833^T^ genes had a large number of inversions and insertions. As shown in Table S3, JCM 10991^T^ and DSM 15833^T^ strains have more mutation sites. The degree of evolution between the JCM 10991^T^ and DSM 15833^T^ strains is different, and there are obvious differences in gene sequence, so we conclude that the two strains are not equivalent strains.

## 3. Materials and Methods

### 3.1. Experimental Strain

*L. equi* IMAU81196 was obtained from Lactic Acid Bacteria Collection Center (LABCC), Key Laboratory of Dairy Biotechnology and Engineering, Ministry of Education, Inner Mongolia Agricultural University. In 2014, *L. equi* IMAU81196 was isolated from the feces collected from foals in Hongyuan County, Sichuan province. The *L. equi* strain IMAU81196 16S ribosomal RNA gene GeneBank sequence number was MG694668. The complete gene information of 12 strains from the genus *Ligilactobacillus*, and three strains of *L. equi* were downloaded from the National Coalition Building Institute (NCBI) website (https://www.ncbi.nlm.nih.gov/ (accessed on 15 May 2021)).

### 3.2. Main Reagents and Instruments

DeMan-Rogosa-Sharpe (MRS) medium broth (Oxoid Co., Ltd., Ireland, UK); TIANamp Bacteria DNA Kit (Tiangen Biochemical Technology Co., Ltd., Beijing, China); automatic autoclave (SX-500 type, Tomy Digital Biology Co., Ltd., Tokyo, Japan); constant-temperature incubator (HWS28 type, Shanghai Yiheng Technology Co., Ltd., Shanghai, China); high-speed centrifuge (5810R, Eppendorf Co., Ltd., Hamburg, Germany); electrophoresis instrument (DYY-12, Beijing Liuyi Instrument Factory, Beijing, China); gel imaging analyzer (CDS8000, Analytik Jena US LLC., Jena, Germany); PCR instrument (PTC-200, Bole Co., Ltd., New York, NY, USA); constant-temperature water bath (HWS28, Shanghai Yiheng Technology Co., Ltd., Shanghai, China); microscope (CX33, Olympus Co., Ltd., Tokyo, Japan).

### 3.3. Strain Culture

Strains stored in ampoules were inoculated into MRS broth and cultured under anaerobic conditions at 37 °C for 24 h for reproduction of the first generation. Strains were continuously subcultured in liquid medium until they reached the third generation. Part of the culture medium of the third generation was collected in 1.5 mL EP tubes, centrifuged at 12,000× *g* for 2 min, and the supernatant was discarded; DNA was extracted from the pellet [47].

Samples from the third-generation bacterial culture were centrifuged at 3800 rmp for 5 min; the supernatant was discarded, and PBS buffer was added. This process was repeated 2–3 times to clean the strain. After Gram staining and microscopic examination, the remaining bacteria were placed in a 15 mL centrifuge tube, quenched with liquid nitrogen, and stored at −80 °C.

### 3.4. DNA Extraction and Genome Sequencing

A bacterial genomic DNA extraction Kit (TIANGEN, Beijing, China) was used to extract the DNA from *L. equi* IMAU81196. DNA fragments were amplified by polymerase chain reaction (PCR), and the 16S rRNA gene was amplified and sequenced by universal primers (the forward primer was 27F (5′-AGAGTTTGACCTGGCTAG -3′), and the reverse primer was 1492R (5′-CTACGGCTCCTTGTTCGA -3′)) [48]. The PCR amplification system and amplification conditions were as described by Yu et al. [49]. After the target strain was identified, its whole gene was sequenced. The 150 bp paired-end (PE) sequencing library was constructed using the Illumina Novaseq 6000 sequencing platform. The average coverage of high-quality data was about 500×.

### 3.5. Genome Assembly of Strains

Initial sequencing data were first filtered and quality evaluated. The software package SOAPdenovo v2.0 was then used to splice and assemble the high-quality reads, and the appropriate Kmer value was selected to splice and assemble filtered data and single base corrections [50]. Genome size, scaffold number, N50 length, N90 length, and GC content were used to evaluate the assembly results. Finally, sequences with good assembly results were selected for subsequent Soap verification. Then, Capcloser software (http://sourceforge.net/projects/soapdenovo2/files/GapCloser/ (accessed on 28 April 2021)) was used to fill gaps and correct single bases to complete gene assembly.

### 3.6. Calculation of Total Nucleotide Consistency (TNI) and Average Nucleotide Consistency (ANI)

The average nucleotide identity (ANI) and total nucleotide identity (TNI) of 16 strains were calculated. Calculations were based on the methods of Goris et al. [19] and Chen et al. [51]. A self-made Perl script was used to evaluate genetic relationships between species. We used TBtools [52] software to draw clustering heat maps.

### 3.7. Analysis of Core Gene Set and Phylogenetic Tree

Prokka software [53] was used to annotate the genes of the 16 strains. Roay software [54] was used to count the core genes. Based on the core gene set, treebest software was used to construct a phylogenetic tree using neighbor-joining (NJ). Itol (https://itol.embl.de/ (accessed on 2 June 2021)) was used to draw the phylogenetic tree online and explore development relationships among strains.

### 3.8. RAST Notes

The nucleic acid sequence files of the four strains of *L. equi* were uploaded to Rapid Annotation using Subsystem Technology (RAST; http://rast.nmpdr.org/rast.cgi (accessed on 8 June 2021)) for annotation.

### 3.9. Analysis of Carbohydrate-Active Enzymes

Gene sequences of the four *L. equi* strains were uploaded to dbcan2 [55] (http://bcb.unl.edu/dbCAN2/ (accessed on 8 June 2021)) for annotation. The gene sequences were annotated with carbohydrate-active enzymes (CAZy). The sequences of the strains were analyzed in combination with data on carbohydrate-active enzymes published on the official website of carbohydrate-active enzymes.

### 3.10. Prediction of Prophage

We used PHASTER software [56] (PHAge Search Tool; http://phast.wishartlab.com/ (accessed on 10 June 2021)) to identify the prophage region in the genome of *L. equi*, locate the prophage sequence, and display the genome characteristics.

### 3.11. Commonality Analysis

Mauve software [57] was used to analyze the genomic sequences of the four strains of *L. equi*. *L. equi* JCM 10991^T^ was used as the reference strain.

## 4. Conclusions

In the present study, the adaptation of *L. equi* was determined by comparative genomic analysis based on genomic data collected for strain IMAU81196 combined with data for three further *L. equi* strains from the NCBI database. We found that *L. equi* has cellulose-degrading enzymes, and each strain contained at least one prophage, which may be the result of strain adaptation to the intestinal environment of horses. *L. equi* JCM 10991^T^ and DSM 15833^T^ were analyzed in terms of genome characteristics, SNP mutation sites, RAST annotations, and phage prediction, and they were determined to be nonequivalent strains. This study enriches the genomic information about *L. equi* and provides reasonable support for follow-up research, development, and utilization of strains. In addition, animal endogenous probiotics are more susceptible to colonization in the intestinal tract, and their cellulose-degrading enzymes produced are more conducive to the digestion and absorption of food in the host intestine. *L. equi* strains can be added to high-fiber animal feed for better digestion and absorption by the host. Therefore, *L.* *equi* has potential as a feed additive.

## Figures and Tables

**Table 1:** Basic genomic characteristics of strains of the genus *Ligilactobacillus*.

Strain	Isolation Source	Genome Size (Mbp)	GC Content (%)	CDS	tRNA	rRNA	Accession NCBI
*L. acidipiscis* DSM 15353^T^	Fermented fish	2.22	39.07	2230	32	2	AZFI01000000
*L. agilis* DSM 20509^T^	Municipal sewage	1.96	41.74	2025	40	3	AYYP01000000
*L. animalis* DSM 20602^T^	Dental plaque of baboon	1.79	41.07	1812	34	4	AYYW01000000
*L. apodemi* DSM 16634^T^	Feces, wild Japanese wood mouse	1.99	38.60	2032	49	4	AZFT01000000
*L. aviarius* DSM 20655^T^	Feces of chicken	1.60	40.12	1585	37	3	AYZA01000000
*L.ceti* DSM 22408^T^	Lungs of a beaked whale	1.33	33.73	1273	51	3	AUHP01000000
*L.hayakitensis* DSM 18933^T^	Feces of horse	1.60	34.03	1583	36	2	AZGD00000000
*L. murinus* DSM 20452^T^	Intestine of rat	2.08	40.03	2040	24	4	AYYN01000000
*L. pobuzihii* NBRC 103219^T^	Pobuzihi (fermented cummingcordia)	2.23	37.70	2121	46	4	JQCN01000000
*L. ruminis* DSM 20403^T^	Bovine rumen	1.94	43.37	1912	40	7	AYYL01000000
*L. saerimneri* DSM 16049^T^	Pig feces	1.64	42.54	1726	35	4	AZFP01000000
*L. salivarius* DSM 20555^T^	Saliva	1.89	32.5	1920	25	4	AYYT01000000
*L. equi* DPC 6820	Feces of horses	2.07	39.21	2077	44	2	AWWH01000000
*L. equi* DSM 15833^T^	Feces of horses	2.18	39.03	2188	50	2	AZFH01000000
*L. equi* JCM 10991^T^	Feces of horses	2.14	38.99	2425	18	4	BAMI01000000
*L. equi* IMAU81196	Feces of horses	1.95	39.48	1905	33	4	JAKGTO000000000

**Table 2:** Specific functional genes of *L. equi* strains.

IMAU81196	Function	JCM 10991^T^	Function	DSM 15833^T^	Function	DPC 6820	Function
*aro*A	3-phosphoshikimate 1-carboxyvinyltransferase	*glt*X	Glutamate—RNA ligase	*fru*A	Fructan beta-fructosidase precursor	*cys*E	Serine acetyltransferase
*aro*C	Chorismate synthase	*ala*S	Alanine—tRNA ligase			*trp*F	*N*-(5’-phosphoribosyl) anthranilate isomerase
*aro*K	Shikimate kinase	*pro*S	Proline—tRNA ligase			*yhd*J	DNA adenine methyltransferase
*bgl*F	PTS system beta-glucoside-specific EIIBCA component	*leu*S	Leucine—tRNA ligase			*psu*K	Pseudouridine kinase
*bgl*H	Aryl-phospho-beta-D-glucosidase	*val*S	Valine—tRNA ligase				
*bgl*G	Cryptic beta-glucoside bgl operon antiterminator	*asp*S	Aspartate—tRNA ligase				
*dha*M	PTS-dependent dihydroxyacetone kinase, phosphotransferase subunit	*arg*S	Arginine—tRNA ligase				
*dha*L	PTS-dependent dihydroxyacetone kinase, ADP-binding subunit	*ile*S	Isoleucine—tRNA ligase				
*dha*K	PTS-dependent dihydroxyacetone kinase, dihydroxyacetone-binding subunit	*cyc*A	D-serine/D-alanine/glycine transporter				
*dha*Q	DhaKLM operon coactivator	*fol*T	Folate transporter				
*dha*S	HTH-type dhaKLM operon transcriptional activator	*znu*B	High-affinity zinc uptake system membrane protein				

**Table 3:** Distribution of the prophage regions among the *L. equi* strains.

Strains	Prophage Region	Completeness	Region Length(kb)	Total Proteins	GC %	Most Common Phage (Number of Genes)
JCM 10991^T^	1	Incomplete	11.60	21	40.00	PHAGE_Lactob_phiPYB5_NC_027982(3)
	2	Questionable	22.40	37	41.53	PHAGE_Lister_B054_NC_009813 (6)
	3	Intact	36.00	62	39.51	PHAGE_Lactob_LF1_NC_019486 (15)
	4	Incomplete	10.90	14	37.55	PHAGE_Clostr_c_st_NC_007581 (2)
DSM 15833^T^	1	Questionable	31.10	15	38.21	PHAGE_Clostr_c_st_NC_007581 (3)
	2	Intact	42.70	63	40.54	PHAGE_Lister_B054_NC_009813 (11)
	3	Intact	38.40	62	39.34	PHAGE_Lactob_LF1_NC_019486 (15)
IMAU81196	1	Intact	14.90	21	34.14	PHAGE_Lactob_JCL1032_NC_019456 (4)
DPC 6820	1	Intact	18.50	19	40.10	PHAGE_Lactob_phig1e_NC_004305 (13)
	2	Questionable	15.80	20	43.33	PHAGE_Lactob_LfeSau_NC_029068 (8)
	3	Incomplete	11.90	20	36.54	PHAGE_Lactob_PLE3_NC_031125 (3)
	4	Questionable	16.0	17	39.84	PHAGE_Lister_LP_101_NC_024387 (8)
	5	Questionable	5.40	10	36.88	PHAGE_Clostr_c_st_NC_007581 (2)
	6	Incomplete	13.90	20	39.65	PHAGE_Lactob_phig1e_NC_004305 (3)

## Data Availability

The whole-genome shotgun sequences of the strain of *L. equi* IMAU81196 have been deposited at DDBJ/EMBL/GenBank under the accession number JAKGTO000000000. The BioProject accession number is PRJNA796805.

## Supplementary Materials

The following supporting information can be downloaded at: https://www.mdpi.com/article/10.3390/molecules27061867/s1, Figure S1: Phylogenetic relationship of 14 strains from the genus *Ligilactobacillus*; Figure S2: Synteny analysis of *L. equi* using the genome of strain JCM 10991^T^ as a reference; Table S1: Genes encoded by carbohydrate metabolism of four strains of *L. equi*; Table S2: Enzymes encoding cellulose degradation; Table S3: SNP site of *L. equi* DSM 15833 (reference strain *L. equi* JCM 10991^T^).
