# Supplementary material for: Comparative Genomic Analysis Reveals Intestinal Habitat Adaptation of Ligilactobacillus equi Rich in Prophage and Degrading Cellulase
Source: Molecules. 2022 Mar 14;27(6):1867. doi: 10.3390/molecules27061867 (PMC8952416; doi:10.3390/molecules27061867)
Supplement: Supplementary file 1 [file molecules-27-01867-s001.zip › molecules-1617461-supplementary.pdf]

Supplementary Material

Comparative Genomic Analysis Reveals Intestinal Habitat Adaptation of *Ligilactobacillus equi* Rich in Prophage and De-grading Cellulase

Yu Li [1,2,3,4], Chen Liu [1,2,3,4], Qing Liu [1,2,3,4] and Wenjun Liu [1,2,3,4,*]

1 Key Laboratory of Dairy Biotechnology and Engineering, Inner Mongolia Agricultural University, Ministry of Education, Hohhot 010018, China; ly401337962@163.com (Y.L.); lc911005xz@163.com (C.L.); liuqing0471@126.com (Q.L.)

2 Key Laboratory of Dairy Products Processing, Inner Mongolia Agricultural University, Ministry of Agriculture and Rural Affairs, Hohhot 010018, China

3 Inner Mongolia Key Laboratory of Dairy Biotechnology and Engineering, Inner Mongolia Agricultural University, Hohhot 010018, China

4 Collaborative Innovative Center of Ministry of Education for Lactic Acid Bacteria and Fermented Dairy Products, Inner Mongolia Agricultural University, Hohhot 010018, China

* Correspondence: wjliu168@163.com; Tel.: +86-471-4308703; Fax: +86-471-4305357

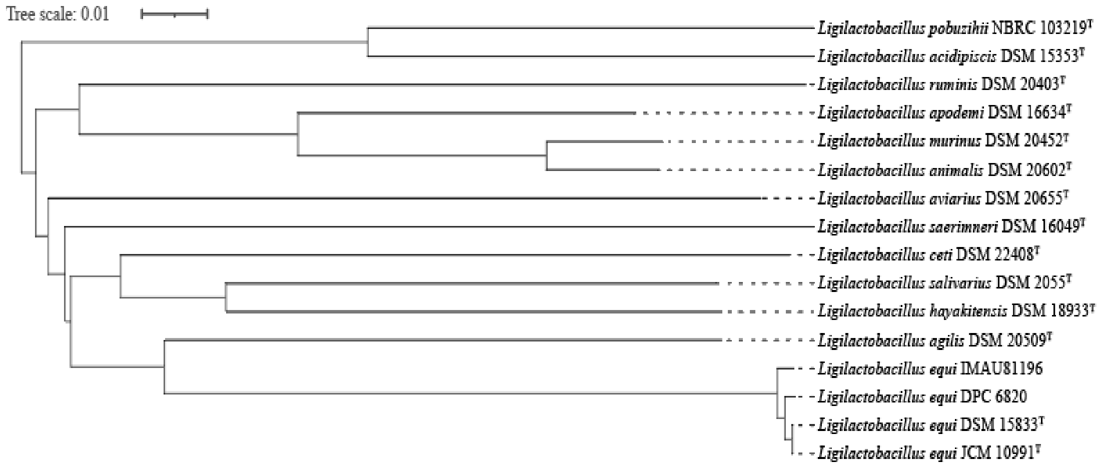

Figure S1. Phylogenetic relationship of 14 strains from the genus *Ligilactobacillus*

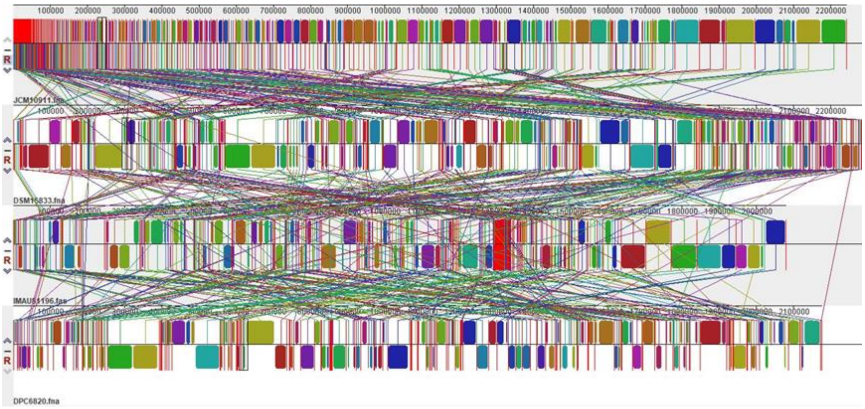

Figure S2. Synteny analysis of *Ligilactobacillus equi* using the genome of strain JCM 10991^{T} as a reference.

Table S1. Genes encoded by carbohydrate metabolism of four strains of *Ligilactobacillus equi*

		IMAU81196	JCM 10991^{T}	DSM 15833	DPC 6820
Central carbohydrate metabolism	Pyruvate metabolism II: acetyl-CoA, acetogenesis from pyruvate	11	13	11	11
	Pyruvate metabolism I: anaplerotic reactions, PEP	5	5	5	5
	Pyruvate Alanine Serine Interconversions	5	7	5	5
	Dehydrogenase complexes	3	4	3	3
	Dihydroxyacetone kinases	5	0	0	0
	Pentose phosphate pathway	13	13	12	0
Aminosugars	Chitin and N-acetylglucosamine utilization	2	2	2	2
Di- and oligosaccharides	Sucrose utilization	7	6	5	5
	Lactose and Galactose Uptake and Utilization	8	9	9	8
One-carbon Metabolism	One-carbon metabolism by tetrahydropterines	4	4	4	4
Organic acids	Glycerate metabolism	6	5	5	4
	Alpha-acetolactate operon	2	3	2	2
Fermentation	Fermentations: Lactate	14	13	12	14
	Acetoin, butanediol metabolism	2	3	2	2
Sugar alcohols	Glycerol and Glycerol-3-phosphate Uptake and Utilization	9	8	7	8
Carbohydrates - no subcategory	VC0266	1	1	1	1
Monosaccharides	Mannose Metabolism	4	4	4	4
	D-ribose utilization	1	1	1	1
	D-galactarate, D-glucarate and D-glycerate catabolism	0	4	4	3
	D-galactarate, D-glucarate and D-glycerate catabolism - gjo	0	4	4	3
	L-Arabinose utilization	0	3	3	0
	Fructose utilization	7	9	7	0
	D-Galacturonate and D-Glucuronate Utilization	11	0	0	0

Table S2. Enzymes encoding cellulose degradation

	Number of enzymes encoded				Encoded enzymes
	IMAU81196	JCM10991	DSM15833	DPC 6820	
GT2	13	10	11	9	Cellulose synthase (EC 2.4.1.12);
					Dolichyl-phosphate β-D-mannosyltransferase (EC 2.4.1.83);
					Chitin oligosaccharide synthase (EC 2.4.1.-)
GH3	2	2	2	0	β-glucosidase (EC 3.2.1.21);
					Xylan 1,4-β-xylosidase(EC 3.2.1.37)
GH43	1	1	1	1	Xylanase(EC 3.2.1.8);
CE4	1	0	0	1	Acetyl xylan esterase (EC 3.1.1.72);
					Chitin deacetylase(EC 3.5.1.41)

Table S3. SNP site of *Ligilactobacillus equi* DSM 15833 (Reference strain *Ligilactobacillus equi* JCM 10991^T)

SNP pattern	JCM 10991^T Contig Number	JCM 109911^T sequence Position In Contg	JCM 109911^T Genome Wide Position	DSM 15833 Contig Number	DSM 15833 sequence Position In Contg	DSM 15833 Genome Wide Position
AG	NZ_BAMI01000155.1	6	1602	NZ_AZFH01000106.1	600	552793
AG	NZ_BAMI01000155.1	9	1605	NZ_AZFH01000106.1	603	552796
AT	NZ_BAMI01000155.1	10	1606	NZ_AZFH01000106.1	604	552797
CT	NZ_BAMI01000154.1	36	2201	NZ_AZFH01000187.1	138	1775938
AG	NZ_BAMI01000154.1	35	2200	NZ_AZFH01000187.1	139	1775939
AG	NZ_BAMI01000154.1	34	2199	NZ_AZFH01000187.1	140	1775940
CA	NZ_BAMI01000154.1	32	2197	NZ_AZFH01000187.1	142	1775942
CA	NZ_BAMI01000154.1	27	2192	NZ_AZFH01000187.1	147	1775947
GA	NZ_BAMI01000154.1	23	2188	NZ_AZFH01000187.1	151	1775951
TC	NZ_BAMI01000154.1	21	2186	NZ_AZFH01000187.1	156	1775956
AT	NZ_BAMI01000154.1	19	2184	NZ_AZFH01000187.1	158	1775958
TG	NZ_BAMI01000154.1	18	2183	NZ_AZFH01000187.1	159	1775959
TA	NZ_BAMI01000154.1	17	2182	NZ_AZFH01000187.1	160	1775960
TC	NZ_BAMI01000154.1	14	2179	NZ_AZFH01000187.1	163	1775963
AG	NZ_BAMI01000154.1	13	2178	NZ_AZFH01000187.1	164	1775964
CT	NZ_BAMI01000154.1	12	2177	NZ_AZFH01000187.1	165	1775965
GA	NZ_BAMI01000154.1	8	2173	NZ_AZFH01000187.1	169	1775969
AG	NZ_BAMI01000154.1	7	2172	NZ_AZFH01000187.1	170	1775970
GT	NZ_BAMI01000154.1	6	2171	NZ_AZFH01000187.1	171	1775971
GC	NZ_BAMI01000154.1	2	2167	NZ_AZFH01000187.1	176	1775976
AC	NZ_BAMI01000154.1	1	2166	NZ_AZFH01000187.1	177	1775977
TG	NZ_BAMI01000155.1	391	1987	NZ_AZFH01000187.1	356	1776156

GA	NZ_BAMI01000155.1	328	1924	NZ_AZFH01000187.1	419	1776219
CT	NZ_BAMI01000152.1	504	3827	NZ_AZFH01000084.1	585	368138
AC	NZ_BAMI01000147.1	3	6392	NZ_AZFH01000043.1	193	2127730
AG	NZ_BAMI01000140.1	28	11352	NZ_AZFH01000077.1	30	202185
AG	NZ_BAMI01000140.1	25	11349	NZ_AZFH01000077.1	36	202191
AC	NZ_BAMI01000140.1	24	11348	NZ_AZFH01000077.1	37	202192
AC	NZ_BAMI01000140.1	23	11347	NZ_AZFH01000077.1	38	202193
AG	NZ_BAMI01000140.1	22	11346	NZ_AZFH01000077.1	39	202194
AT	NZ_BAMI01000140.1	21	11345	NZ_AZFH01000077.1	40	202195
AG	NZ_BAMI01000140.1	19	11343	NZ_AZFH01000077.1	42	202197
AT	NZ_BAMI01000140.1	15	11339	NZ_AZFH01000077.1	46	202201
CA	NZ_BAMI01000140.1	13	11337	NZ_AZFH01000077.1	48	202203
AG	NZ_BAMI01000140.1	10	11334	NZ_AZFH01000077.1	51	202206
GA	NZ_BAMI01000140.1	3	11327	NZ_AZFH01000077.1	54	202209
AC	NZ_BAMI01000140.1	2	11326	NZ_AZFH01000077.1	55	202210
AT	NZ_BAMI01000140.1	1	11325	NZ_AZFH01000077.1	56	202211
TC	NZ_BAMI01000140.1	115	11439	NZ_AZFH01000089.1	112	431811
GA	NZ_BAMI01000139.1	317	12515	NZ_AZFH01000045.1	276	2141104
CT	NZ_BAMI01000139.1	320	12518	NZ_AZFH01000045.1	279	2141107
TC	NZ_BAMI01000138.1	826	13979	NZ_AZFH01000142.1	695	1146839
TG	NZ_BAMI01000131.1	965	21427	NZ_AZFH01000079.1	509	203785
AG	NZ_BAMI01000125.1	356	27907	NZ_AZFH01000172.1	357	1488850
TC	NZ_BAMI01000125.1	515	28066	NZ_AZFH01000172.1	516	1489009
TC	NZ_BAMI01000119.1	1024	36951	NZ_AZFH01000070.1	762	157521
AT	NZ_BAMI01000118.1	701	38176	NZ_AZFH01000076.1	805	201300
TC	NZ_BAMI01000118.1	1134	38609	NZ_AZFH01000076.1	1238	201733

GT	NZ_BAMI01000117.1	188	39219	NZ_AZFH01000006.1	193	2255308
GA	NZ_BAMI01000117.1	190	39221	NZ_AZFH01000006.1	195	2255310
CT	NZ_BAMI01000117.1	193	39224	NZ_AZFH01000006.1	198	2255313
GA	NZ_BAMI01000117.1	195	39226	NZ_AZFH01000006.1	200	2255315
CA	NZ_BAMI01000117.1	204	39235	NZ_AZFH01000006.1	209	2255324
TC	NZ_BAMI01000117.1	205	39236	NZ_AZFH01000006.1	210	2255325
TA	NZ_BAMI01000117.1	207	39238	NZ_AZFH01000006.1	212	2255327
CA	NZ_BAMI01000117.1	208	39239	NZ_AZFH01000006.1	213	2255328
TC	NZ_BAMI01000117.1	209	39240	NZ_AZFH01000006.1	214	2255329
CG	NZ_BAMI01000117.1	210	39241	NZ_AZFH01000006.1	215	2255330
TA	NZ_BAMI01000117.1	212	39243	NZ_AZFH01000006.1	217	2255332
CT	NZ_BAMI01000117.1	213	39244	NZ_AZFH01000006.1	218	2255333
TG	NZ_BAMI01000117.1	214	39245	NZ_AZFH01000006.1	219	2255334
AG	NZ_BAMI01000117.1	215	39246	NZ_AZFH01000006.1	220	2255335
TG	NZ_BAMI01000117.1	217	39248	NZ_AZFH01000006.1	222	2255337
TA	NZ_BAMI01000117.1	218	39249	NZ_AZFH01000006.1	223	2255338
TA	NZ_BAMI01000117.1	220	39251	NZ_AZFH01000006.1	225	2255340
GA	NZ_BAMI01000117.1	222	39253	NZ_AZFH01000006.1	227	2255342
AT	NZ_BAMI01000117.1	223	39254	NZ_AZFH01000006.1	228	2255343
GA	NZ_BAMI01000117.1	225	39256	NZ_AZFH01000006.1	230	2255345
AG	NZ_BAMI01000117.1	231	39262	NZ_AZFH01000006.1	234	2255349
TC	NZ_BAMI01000117.1	235	39266	NZ_AZFH01000006.1	238	2255353
TC	NZ_BAMI01000117.1	239	39270	NZ_AZFH01000006.1	241	2255356
TG	NZ_BAMI01000117.1	243	39274	NZ_AZFH01000006.1	245	2255360
TA	NZ_BAMI01000117.1	248	39279	NZ_AZFH01000006.1	247	2255362
AG	NZ_BAMI01000117.1	253	39284	NZ_AZFH01000006.1	252	2255367

CT	NZ_BAMI01000117.1	255	39286	NZ_AZFH01000006.1	254	2255369
TC	NZ_BAMI01000117.1	256	39287	NZ_AZFH01000006.1	261	2255376
TC	NZ_BAMI01000117.1	260	39291	NZ_AZFH01000006.1	265	2255380
TA	NZ_BAMI01000117.1	262	39293	NZ_AZFH01000006.1	267	2255382
GC	NZ_BAMI01000117.1	263	39294	NZ_AZFH01000006.1	268	2255383
GA	NZ_BAMI01000117.1	264	39295	NZ_AZFH01000006.1	269	2255384
CA	NZ_BAMI01000117.1	265	39296	NZ_AZFH01000006.1	270	2255385
CT	NZ_BAMI01000117.1	267	39298	NZ_AZFH01000006.1	272	2255387
TA	NZ_BAMI01000117.1	268	39299	NZ_AZFH01000006.1	273	2255388
TC	NZ_BAMI01000117.1	269	39300	NZ_AZFH01000006.1	274	2255389
TG	NZ_BAMI01000117.1	271	39302	NZ_AZFH01000006.1	276	2255391
AG	NZ_BAMI01000117.1	274	39305	NZ_AZFH01000006.1	279	2255394
AT	NZ_BAMI01000117.1	275	39306	NZ_AZFH01000006.1	280	2255395
AG	NZ_BAMI01000117.1	277	39308	NZ_AZFH01000006.1	282	2255397
TC	NZ_BAMI01000117.1	278	39309	NZ_AZFH01000006.1	283	2255398
GA	NZ_BAMI01000117.1	279	39310	NZ_AZFH01000006.1	284	2255399
TC	NZ_BAMI01000117.1	280	39311	NZ_AZFH01000006.1	285	2255400
TC	NZ_BAMI01000117.1	281	39312	NZ_AZFH01000006.1	286	2255401
GA	NZ_BAMI01000117.1	286	39317	NZ_AZFH01000006.1	295	2255410
TG	NZ_BAMI01000117.1	292	39323	NZ_AZFH01000006.1	299	2255414
GC	NZ_BAMI01000117.1	294	39325	NZ_AZFH01000006.1	301	2255416
AC	NZ_BAMI01000117.1	1331	40362	NZ_AZFH01000058.1	1038	2256454
TC	NZ_BAMI01000114.1	1389	45235	NZ_AZFH01000040.1	42	1893644
TC	NZ_BAMI01000107.1	2274	59791	NZ_AZFH01000175.1	154	1518314
AG	NZ_BAMI01000103.1	335	68097	NZ_AZFH01000046.1	387	2141531
TC	NZ_BAMI01000100.1	1	75936	NZ_AZFH01000091.1	512	432743

GA	NZ_BAMI01000098.1	2728	84751	NZ_AZFH01000028.1	528	1416009
AG	NZ_BAMI01000098.1	57	82080	NZ_AZFH01000029.1	1	1418683
TC	NZ_BAMI01000097.1	329	85606	NZ_AZFH01000005.1	4449	2171462
AN	NZ_BAMI01000095.1	3443	95466	NZ_AZFH01000116.1	344	879918
TC	NZ_BAMI01000095.1	1873	93896	NZ_AZFH01000116.1	1914	881488
TC	NZ_BAMI01000095.1	1358	93381	NZ_AZFH01000116.1	2430	882004
AG	NZ_BAMI01000096.1	3463	92022	NZ_AZFH01000116.1	3791	883365
TC	NZ_BAMI01000096.1	3459	92018	NZ_AZFH01000116.1	3797	883371
AG	NZ_BAMI01000093.1	462	99875	NZ_AZFH01000204.1	31325	2098277
TG	NZ_BAMI01000093.1	494	99907	NZ_AZFH01000204.1	31357	2098309
TG	NZ_BAMI01000093.1	495	99908	NZ_AZFH01000204.1	31358	2098310
AC	NZ_BAMI01000093.1	926	100339	NZ_AZFH01000204.1	31789	2098741
GC	NZ_BAMI01000092.1	229	103634	NZ_AZFH01000149.1	4075	1155293
TC	NZ_BAMI01000083.1	5005	152244	NZ_AZFH01000203.1	732	2062013
TN	NZ_BAMI01000083.1	4752	151991	NZ_AZFH01000203.1	985	2062266
AN	NZ_BAMI01000083.1	4751	151990	NZ_AZFH01000203.1	986	2062267
GN	NZ_BAMI01000083.1	4750	151989	NZ_AZFH01000203.1	987	2062268
CN	NZ_BAMI01000083.1	4749	151988	NZ_AZFH01000203.1	988	2062269
AN	NZ_BAMI01000083.1	4748	151987	NZ_AZFH01000203.1	989	2062270
AN	NZ_BAMI01000083.1	4747	151986	NZ_AZFH01000203.1	990	2062271
AN	NZ_BAMI01000083.1	4746	151985	NZ_AZFH01000203.1	991	2062272
AN	NZ_BAMI01000083.1	4745	151984	NZ_AZFH01000203.1	992	2062273
GN	NZ_BAMI01000083.1	4744	151983	NZ_AZFH01000203.1	993	2062274
CN	NZ_BAMI01000083.1	4743	151982	NZ_AZFH01000203.1	994	2062275
CN	NZ_BAMI01000083.1	4742	151981	NZ_AZFH01000203.1	995	2062276
CN	NZ_BAMI01000083.1	4741	151980	NZ_AZFH01000203.1	996	2062277

CN	NZ_BAMI01000083.1	4740	151979	NZ_AZFH01000203.1	997	2062278
GN	NZ_BAMI01000083.1	4739	151978	NZ_AZFH01000203.1	998	2062279
CN	NZ_BAMI01000083.1	4738	151977	NZ_AZFH01000203.1	999	2062280
AN	NZ_BAMI01000083.1	4737	151976	NZ_AZFH01000203.1	1000	2062281
CN	NZ_BAMI01000083.1	4736	151975	NZ_AZFH01000203.1	1001	2062282
AN	NZ_BAMI01000083.1	4735	151974	NZ_AZFH01000203.1	1002	2062283
AN	NZ_BAMI01000083.1	4734	151973	NZ_AZFH01000203.1	1003	2062284
AN	NZ_BAMI01000083.1	4733	151972	NZ_AZFH01000203.1	1004	2062285
AG	NZ_BAMI01000083.1	4314	151553	NZ_AZFH01000203.1	1303	2062584
AG	NZ_BAMI01000078.1	3857	182333	NZ_AZFH01000039.1	3464	1888539
GA	NZ_BAMI01000077.1	313	186056	NZ_AZFH01000164.1	324	1314144
CT	NZ_BAMI01000077.1	1310	187053	NZ_AZFH01000071.1	435	158156
GT	NZ_BAMI01000077.1	1349	187092	NZ_AZFH01000072.1	1	158195
AG	NZ_BAMI01000077.1	7266	193009	NZ_AZFH01000117.1	7480	891121
GT	NZ_BAMI01000077.1	7265	193008	NZ_AZFH01000117.1	7481	891122
AG	NZ_BAMI01000077.1	7257	193000	NZ_AZFH01000117.1	7485	891126
TG	NZ_BAMI01000077.1	7254	192997	NZ_AZFH01000117.1	7488	891129
TC	NZ_BAMI01000077.1	7253	192996	NZ_AZFH01000117.1	7489	891130
TG	NZ_BAMI01000077.1	7246	192989	NZ_AZFH01000117.1	7495	891136
TG	NZ_BAMI01000077.1	7240	192983	NZ_AZFH01000117.1	7501	891142
AT	NZ_BAMI01000077.1	7239	192982	NZ_AZFH01000117.1	7502	891143
TC	NZ_BAMI01000077.1	7238	192981	NZ_AZFH01000117.1	7503	891144
AC	NZ_BAMI01000077.1	7237	192980	NZ_AZFH01000117.1	7504	891145
GC	NZ_BAMI01000077.1	7236	192979	NZ_AZFH01000117.1	7505	891146
AC	NZ_BAMI01000077.1	7234	192977	NZ_AZFH01000117.1	7507	891148
TG	NZ_BAMI01000077.1	7233	192976	NZ_AZFH01000117.1	7508	891149

GC	NZ_BAMI01000077.1	7232	192975	NZ_AZFH01000117.1	7509	891150
TA	NZ_BAMI01000077.1	7230	192973	NZ_AZFH01000117.1	7511	891152
AG	NZ_BAMI01000077.1	7227	192970	NZ_AZFH01000117.1	7514	891155
TG	NZ_BAMI01000077.1	7226	192969	NZ_AZFH01000117.1	7515	891156
CG	NZ_BAMI01000077.1	7225	192968	NZ_AZFH01000117.1	7516	891157
AT	NZ_BAMI01000077.1	7223	192966	NZ_AZFH01000117.1	7518	891159
AG	NZ_BAMI01000077.1	7222	192965	NZ_AZFH01000117.1	7519	891160
AG	NZ_BAMI01000077.1	7219	192962	NZ_AZFH01000117.1	7522	891163
GA	NZ_BAMI01000077.1	7218	192961	NZ_AZFH01000117.1	7523	891164
TG	NZ_BAMI01000077.1	7217	192960	NZ_AZFH01000117.1	7524	891165
CG	NZ_BAMI01000077.1	7215	192958	NZ_AZFH01000117.1	7526	891167
TC	NZ_BAMI01000077.1	7214	192957	NZ_AZFH01000117.1	7527	891168
AC	NZ_BAMI01000077.1	7213	192956	NZ_AZFH01000117.1	7528	891169
AG	NZ_BAMI01000077.1	7212	192955	NZ_AZFH01000117.1	7529	891170
TC	NZ_BAMI01000077.1	7211	192954	NZ_AZFH01000117.1	7530	891171
AG	NZ_BAMI01000077.1	7210	192953	NZ_AZFH01000117.1	7531	891172
CA	NZ_BAMI01000077.1	7209	192952	NZ_AZFH01000117.1	7532	891173
AG	NZ_BAMI01000074.1	1798	209889	NZ_AZFH01000097.1	7110	494958
CT	NZ_BAMI01000072.1	8096	231649	NZ_AZFH01000010.1	77205	296988
CT	NZ_BAMI01000072.1	8098	231651	NZ_AZFH01000010.1	77207	296990
CA	NZ_BAMI01000062.1	1	314378	NZ_AZFH01000202.1	72295	2050330
AG	NZ_BAMI01000060.1	11521	348508	NZ_AZFH01000197.1	322	1895925
AG	NZ_BAMI01000060.1	11458	348445	NZ_AZFH01000197.1	385	1895988
TC	NZ_BAMI01000060.1	9271	346258	NZ_AZFH01000197.1	2572	1898175
CT	NZ_BAMI01000058.1	5	360497	NZ_AZFH01000007.1	5308	5308
TA	NZ_BAMI01000056.1	12741	398586	NZ_AZFH01000202.1	54714	2032749

TC	NZ_BAMI01000055.1	170	399437	NZ_AZFH01000096.1	20484	482794
GN	NZ_BAMI01000054.1	4872	417642	NZ_AZFH01000031.1	9558	1438943
GN	NZ_BAMI01000054.1	4871	417641	NZ_AZFH01000031.1	9559	1438944
GN	NZ_BAMI01000054.1	4870	417640	NZ_AZFH01000031.1	9560	1438945
CN	NZ_BAMI01000054.1	4869	417639	NZ_AZFH01000031.1	9561	1438946
CN	NZ_BAMI01000054.1	4868	417638	NZ_AZFH01000031.1	9562	1438947
GN	NZ_BAMI01000054.1	4867	417637	NZ_AZFH01000031.1	9563	1438948
TN	NZ_BAMI01000054.1	4866	417636	NZ_AZFH01000031.1	9564	1438949
TN	NZ_BAMI01000054.1	4865	417635	NZ_AZFH01000031.1	9565	1438950
AN	NZ_BAMI01000054.1	4864	417634	NZ_AZFH01000031.1	9566	1438951
TN	NZ_BAMI01000054.1	4863	417633	NZ_AZFH01000031.1	9567	1438952
TC	NZ_BAMI01000051.1	10940	467294	NZ_AZFH01000204.1	45843	2112795
GT	NZ_BAMI01000051.1	10949	467303	NZ_AZFH01000204.1	45852	2112804
TC	NZ_BAMI01000051.1	10951	467305	NZ_AZFH01000204.1	45854	2112806
TC	NZ_BAMI01000051.1	11088	467442	NZ_AZFH01000204.1	45991	2112943
GA	NZ_BAMI01000049.1	8704	495047	NZ_AZFH01000047.1	8729	2152676
AT	NZ_BAMI01000048.1	14986	516535	NZ_AZFH01000113.1	1208	833170
TC	NZ_BAMI01000048.1	7233	508782	NZ_AZFH01000113.1	8962	840924
CT	NZ_BAMI01000048.1	7214	508763	NZ_AZFH01000113.1	8981	840943
CT	NZ_BAMI01000048.1	7211	508760	NZ_AZFH01000113.1	8984	840946
TA	NZ_BAMI01000048.1	2443	503992	NZ_AZFH01000113.1	13735	845697
CG	NZ_BAMI01000047.1	10569	527344	NZ_AZFH01000051.1	4831	2176547
GA	NZ_BAMI01000047.1	10568	527343	NZ_AZFH01000051.1	4832	2176548
AC	NZ_BAMI01000047.1	10565	527340	NZ_AZFH01000051.1	4835	2176551
TG	NZ_BAMI01000044.1	16582	580548	NZ_AZFH01000202.1	72193	2050228
AC	NZ_BAMI01000043.1	1	580637	NZ_AZFH01000202.1	72282	2050317

CN	NZ_BAMI01000038.1	414	671130	NZ_AZFH01000202.1	23383	2001418
CN	NZ_BAMI01000038.1	415	671131	NZ_AZFH01000202.1	23384	2001419
AN	NZ_BAMI01000038.1	416	671132	NZ_AZFH01000202.1	23385	2001420
AN	NZ_BAMI01000038.1	417	671133	NZ_AZFH01000202.1	23386	2001421
GN	NZ_BAMI01000038.1	418	671134	NZ_AZFH01000202.1	23387	2001422
GN	NZ_BAMI01000038.1	419	671135	NZ_AZFH01000202.1	23388	2001423
TN	NZ_BAMI01000038.1	420	671136	NZ_AZFH01000202.1	23389	2001424
TN	NZ_BAMI01000038.1	421	671137	NZ_AZFH01000202.1	23390	2001425
GN	NZ_BAMI01000038.1	422	671138	NZ_AZFH01000202.1	23391	2001426
GN	NZ_BAMI01000038.1	423	671139	NZ_AZFH01000202.1	23392	2001427
CG	NZ_BAMI01000035.1	21460	751816	NZ_AZFH01000013.1	22270	460986
AG	NZ_BAMI01000035.1	21458	751814	NZ_AZFH01000013.1	22272	460988
AG	NZ_BAMI01000035.1	21457	751813	NZ_AZFH01000013.1	22273	460989
GA	NZ_BAMI01000035.1	21456	751812	NZ_AZFH01000013.1	22274	460990
CT	NZ_BAMI01000030.1	14659	857345	NZ_AZFH01000022.1	48609	1083471
CT	NZ_BAMI01000029.1	24429	891316	NZ_AZFH01000112.1	1	792279
CT	NZ_BAMI01000029.1	24430	891317	NZ_AZFH01000112.1	2	792280
CA	NZ_BAMI01000029.1	24431	891318	NZ_AZFH01000112.1	3	792281
CG	NZ_BAMI01000029.1	24433	891320	NZ_AZFH01000112.1	5	792283
TG	NZ_BAMI01000029.1	24435	891322	NZ_AZFH01000112.1	7	792285
TA	NZ_BAMI01000029.1	24436	891323	NZ_AZFH01000112.1	8	792286
TA	NZ_BAMI01000029.1	24440	891327	NZ_AZFH01000112.1	12	792290
AT	NZ_BAMI01000029.1	24441	891328	NZ_AZFH01000112.1	13	792291
AG	NZ_BAMI01000029.1	24442	891329	NZ_AZFH01000112.1	14	792292
AC	NZ_BAMI01000029.1	24446	891333	NZ_AZFH01000112.1	18	792296
CG	NZ_BAMI01000027.1	17335	933323	NZ_AZFH01000069.1	10945	138944

AG	NZ_BAMI01000026.1	67	944275	NZ_AZFH01000159.1	28864	1276943
CT	NZ_BAMI01000026.1	6	944214	NZ_AZFH01000159.1	28925	1277004
GN	NZ_BAMI01000024.1	1821	1003830	NZ_AZFH01000087.1	1879	403566
GN	NZ_BAMI01000024.1	1822	1003831	NZ_AZFH01000087.1	1880	403567
CN	NZ_BAMI01000024.1	1823	1003832	NZ_AZFH01000087.1	1881	403568
GN	NZ_BAMI01000024.1	1824	1003833	NZ_AZFH01000087.1	1882	403569
TN	NZ_BAMI01000024.1	1825	1003834	NZ_AZFH01000087.1	1883	403570
AN	NZ_BAMI01000024.1	1826	1003835	NZ_AZFH01000087.1	1884	403571
TN	NZ_BAMI01000024.1	1827	1003836	NZ_AZFH01000087.1	1885	403572
TN	NZ_BAMI01000024.1	1828	1003837	NZ_AZFH01000087.1	1886	403573
GN	NZ_BAMI01000024.1	1829	1003838	NZ_AZFH01000087.1	1887	403574
TN	NZ_BAMI01000024.1	1830	1003839	NZ_AZFH01000087.1	1888	403575
GN	NZ_BAMI01000024.1	1831	1003840	NZ_AZFH01000087.1	1889	403576
AN	NZ_BAMI01000024.1	1832	1003841	NZ_AZFH01000087.1	1890	403577
AN	NZ_BAMI01000024.1	1833	1003842	NZ_AZFH01000087.1	1891	403578
TN	NZ_BAMI01000024.1	1834	1003843	NZ_AZFH01000087.1	1892	403579
AN	NZ_BAMI01000024.1	1835	1003844	NZ_AZFH01000087.1	1893	403580
AN	NZ_BAMI01000024.1	1836	1003845	NZ_AZFH01000087.1	1894	403581
GN	NZ_BAMI01000024.1	1837	1003846	NZ_AZFH01000087.1	1895	403582
CN	NZ_BAMI01000024.1	1838	1003847	NZ_AZFH01000087.1	1896	403583
AN	NZ_BAMI01000024.1	1839	1003848	NZ_AZFH01000087.1	1897	403584
CN	NZ_BAMI01000024.1	1840	1003849	NZ_AZFH01000087.1	1898	403585
TN	NZ_BAMI01000024.1	1841	1003850	NZ_AZFH01000087.1	1899	403586
AN	NZ_BAMI01000024.1	1842	1003851	NZ_AZFH01000087.1	1900	403587
CN	NZ_BAMI01000024.1	1843	1003852	NZ_AZFH01000087.1	1901	403588
GN	NZ_BAMI01000024.1	1844	1003853	NZ_AZFH01000087.1	1910	403597

GN	NZ_BAMI01000024.1	1845	1003854	NZ_AZFH01000087.1	1911	403598
CN	NZ_BAMI01000024.1	1846	1003855	NZ_AZFH01000087.1	1912	403599
AN	NZ_BAMI01000024.1	1847	1003856	NZ_AZFH01000087.1	1913	403600
AN	NZ_BAMI01000024.1	1848	1003857	NZ_AZFH01000087.1	1914	403601
TN	NZ_BAMI01000024.1	1849	1003858	NZ_AZFH01000087.1	1915	403602
AN	NZ_BAMI01000024.1	1850	1003859	NZ_AZFH01000087.1	1916	403603
AN	NZ_BAMI01000024.1	1851	1003860	NZ_AZFH01000087.1	1917	403604
TN	NZ_BAMI01000024.1	1852	1003861	NZ_AZFH01000087.1	1918	403605
AN	NZ_BAMI01000024.1	1853	1003862	NZ_AZFH01000087.1	1919	403606
GN	NZ_BAMI01000024.1	1854	1003863	NZ_AZFH01000087.1	1920	403607
CN	NZ_BAMI01000024.1	1855	1003864	NZ_AZFH01000087.1	1921	403608
CN	NZ_BAMI01000024.1	1856	1003865	NZ_AZFH01000087.1	1922	403609
AN	NZ_BAMI01000024.1	1857	1003866	NZ_AZFH01000087.1	1923	403610
AN	NZ_BAMI01000024.1	1858	1003867	NZ_AZFH01000087.1	1924	403611
TN	NZ_BAMI01000024.1	1859	1003868	NZ_AZFH01000087.1	1925	403612
CN	NZ_BAMI01000024.1	1860	1003869	NZ_AZFH01000087.1	1926	403613
CN	NZ_BAMI01000024.1	1861	1003870	NZ_AZFH01000087.1	1927	403614
TN	NZ_BAMI01000024.1	1862	1003871	NZ_AZFH01000087.1	1928	403615
CN	NZ_BAMI01000024.1	1863	1003872	NZ_AZFH01000087.1	1929	403616
TN	NZ_BAMI01000024.1	1864	1003873	NZ_AZFH01000087.1	1930	403617
CN	NZ_BAMI01000024.1	1865	1003874	NZ_AZFH01000087.1	1931	403618
AN	NZ_BAMI01000024.1	1866	1003875	NZ_AZFH01000087.1	1932	403619
TC	NZ_BAMI01000024.1	17039	1019048	NZ_AZFH01000087.1	17107	418794
GA	NZ_BAMI01000020.1	29964	1152439	NZ_AZFH01000192.1	30028	1883994
CT	NZ_BAMI01000020.1	29973	1152448	NZ_AZFH01000192.1	30037	1884003
GT	NZ_BAMI01000020.1	29998	1152473	NZ_AZFH01000192.1	30062	1884028

TA	NZ_BAMI01000019.1	21610	1174805	NZ_AZFH01000157.1	22833	1235834
TN	NZ_BAMI01000016.1	42	1255523	NZ_AZFH01000138.1	37000	1033244
GN	NZ_BAMI01000016.1	41	1255522	NZ_AZFH01000138.1	37001	1033245
GN	NZ_BAMI01000016.1	40	1255521	NZ_AZFH01000138.1	37002	1033246
GN	NZ_BAMI01000016.1	39	1255520	NZ_AZFH01000138.1	37003	1033247
CN	NZ_BAMI01000016.1	38	1255519	NZ_AZFH01000138.1	37004	1033248
GN	NZ_BAMI01000016.1	37	1255518	NZ_AZFH01000138.1	37005	1033249
TN	NZ_BAMI01000016.1	36	1255517	NZ_AZFH01000138.1	37006	1033250
AN	NZ_BAMI01000016.1	35	1255516	NZ_AZFH01000138.1	37007	1033251
TN	NZ_BAMI01000016.1	34	1255515	NZ_AZFH01000138.1	37008	1033252
TN	NZ_BAMI01000016.1	33	1255514	NZ_AZFH01000138.1	37009	1033253
AT	NZ_BAMI01000012.1	38068	1445763	NZ_AZFH01000155.1	5995	1172199
GT	NZ_BAMI01000012.1	38040	1445735	NZ_AZFH01000155.1	6021	1172225
AG	NZ_BAMI01000012.1	38039	1445734	NZ_AZFH01000155.1	6022	1172226
GA	NZ_BAMI01000010.1	3864	1505324	NZ_AZFH01000139.1	3915	1096987
TG	NZ_BAMI01000009.1	25781	1579437	NZ_AZFH01000037.1	34356	1810902
GT	NZ_BAMI01000009.1	25783	1579439	NZ_AZFH01000037.1	34358	1810904
CG	NZ_BAMI01000009.1	25786	1579442	NZ_AZFH01000037.1	34361	1810907
TC	NZ_BAMI01000009.1	25787	1579443	NZ_AZFH01000037.1	34362	1810908
CT	NZ_BAMI01000008.1	56759	1663651	NZ_AZFH01000011.1	168	308983
CT	NZ_BAMI01000008.1	56760	1663652	NZ_AZFH01000011.1	169	308984
GT	NZ_BAMI01000008.1	56761	1663653	NZ_AZFH01000011.1	170	308985
AG	NZ_BAMI01000004.1	71843	1918437	NZ_AZFH01000067.1	221	25728
TC	NZ_BAMI01000003.1	59970	1978563	NZ_AZFH01000010.1	17228	237011
TN	NZ_BAMI01000003.1	59154	1977747	NZ_AZFH01000010.1	18029	237812
TN	NZ_BAMI01000003.1	59153	1977746	NZ_AZFH01000010.1	18030	237813

AN	NZ_BAMI01000003.1	59152	1977745	NZ_AZFH01000010.1	18031	237814
AN	NZ_BAMI01000003.1	59151	1977744	NZ_AZFH01000010.1	18032	237815
AN	NZ_BAMI01000003.1	59150	1977743	NZ_AZFH01000010.1	18033	237816
TN	NZ_BAMI01000003.1	59149	1977742	NZ_AZFH01000010.1	18034	237817
GT	NZ_BAMI01000002.1	6	1995747	NZ_AZFH01000181.1	49819	1579296
CA	NZ_BAMI01000001.1	91296	2165431	NZ_AZFH01000001.1	77315	647155
AG	NZ_BAMI01000001.1	22	2074157	NZ_AZFH01000001.1	168613	738453
